# Intravenous iron for heart failure, iron deficiency definitions, and clinical response: the IRONMAN trial

**DOI:** 10.1093/eurheartj/ehae086

**Published:** 2024-03-06

**Authors:** John G F Cleland, Philip A Kalra, Pierpaolo Pellicori, Fraser J Graham, Paul W X Foley, Iain B Squire, Peter J Cowburn, Alison Seed, Andrew L Clark, Ben Szwejkowski, Prithwish Banerjee, Justin Cooke, Mark Francis, Piers Clifford, Aaron Wong, Colin Petrie, John J V McMurray, Elizabeth A Thomson, Kirsty Wetherall, Michele Robertson, Ian Ford, Paul R Kalra, the IRONMAN Study Group, Paul Kalra, Elena Cowan, Charlotte Turner, Rosalynn Austin, Rebeca Lane, Paula Rogers, Paul Foley, Badri Chandrasekaran, Eva Fraile, Lynsey Kyeremeh, Fozia Ahmed, Mark Petrie, Lorraine McGregor, Joanna Osmanska, Fraser Graham, Ninian Lang, Barbara Meyer, Faheem Ahmad, Joanna Osmanska, Iain Squire, Jude Fisher, Philip Kalra, Christina Summersgill, Katarzyna Adeniji, Rajkumar Chinnadurai, Andrew Ludman, Lisa Massimo, Clare Hardman, Daisy Sykes, Peter Cowburn, Sarah Frank, Simon Smith, Alan Japp, Mohamed Anwar, Beth Whittington, Alison Seed, Robin Ray, Vennessa Sookhoo, Sinead Lyons, Abdallah Al-Mohammad, Janet Middle, Kay Housley, Andrew Clark, Jeanne Bulemfu, Christopher Critoph, Victor Chong, Stephen Wood, Benjamin Szwejkowski, Chim Lang, Jackie Duff, Susan MacDonald, Rebekah Schiff, Patrick Donnelly, Thuraia Nageh, Swapna Kunhunny, Mark Petrie, Roy Gardner, Marion McAdam, Elizabeth McPherson, Prithwish Banerjee, Eleanor Sear, Nigel Edwards, Jason Glover, Pierpaolo Pellicori, Clare Murphy, Justin Cooke, Charles Spencer, Mark Francis, Iain Matthews, Hayley McKie, Andrew Marshall, Janet Large, Jenny Stratford, Piers Clifford, Christopher Boos, Philip Keeling, Aaron Wong, Deborah Jones, Alex James, Rhys Williams, Stephen Leslie, Jim Finlayson, Piers Clifford, Andrew Hannah, Philip Campbell, John Walsh, Jane Quinn, Callum Chapman, Susan Piper, Sheetal Patale, Preeti Gupta, Victor Sim, Lucy Knibbs, Kristopher Lyons, Lana Dixon, Colin Petrie, Yuk-ki Wong, Catherine Labinjoh, Simon Duckett, Ian Massey, Henry Savage, Sofia Matias, Jonaifah Ramirez, Charlotte Manisty, Ifza Hussain, Rajiv Sankaranarayanan, Gershan Davis, Samuel McClure, John Baxter, Eleanor Wicks, Jolanta Sobolewska, Jerry Murphy, Ahmed Elzayat, Jay Wright, Simon Williams, Amal Muthumala, Parminder Chaggar, Sue Webber, Gethin Ellis, Mandie Welch, Sudantha Bulugahapitiya, Thomas Jackson, Tapesh Pakrashi, Ameet Bakhai, Vinodh Krishnamurthy, Reto Gamma, Susan Ellery, Charlotte Manisty, Geraint Jenkins, Angus Nightingale, Elizabeth Thomson, Ian Ford, Michele Robertson, Nicola Greenlaw, Kirsty Wetherall, Ross Clarke, Christopher Graham, Sharon Kean, Alan Stevenson, Robbie Wilson, Sarah Boyle, John McHugh, Lisa Hall, Joanne Woollard, Claire Brunton, Eleanor Dinnett, Amanda Reid, Serena Howe, Jill Nicholls, Anna Cunnington, Elizabeth Douglas, Margaret Fegen, Marc Jones, Sheila McGowan, Barbara Ross, Pamela Sandu, Pamela Surtees, Debra Stuart, Nicholas Boon, Shannon Amoils, Callum Chapman, John Cleland, Thomas Goldin Diness, Ian Ford, Paul Kalra, Philip Kalra, Iain Macdougall, John McMurray, Richard Mindham, Mark Petrie, Pamela Sandu, Iain Squire, Claes Christian Strom, Elizabeth Thomson, Maureen Travers, Robert Wilcox, Allan Struthers, Patrick Mark, Christopher Weir, John Cleland, Fraser Graham, Pierpaolo Pellicori

**Affiliations:** School of Cardiovascular and Metabolic Health, University of Glasgow, 126 University Place, Glasgow, Lanarkshire, G12 8TA, UK; Salford Royal Hospital, Northern Care Alliance NHS Foundation Trust, Salford, UK; The University of Manchester, Manchester, UK; School of Cardiovascular and Metabolic Health, University of Glasgow, 126 University Place, Glasgow, Lanarkshire, G12 8TA, UK; School of Cardiovascular and Metabolic Health, University of Glasgow, 126 University Place, Glasgow, Lanarkshire, G12 8TA, UK; Great Western Hospitals NHS Foundation Trust, Swindon, UK; Department of Cardiovascular Sciences, University of Leicester, Leicester, UK; University Hospital Southampton NHS Foundation Trust, Southampton, UK; Blackpool Teaching Hospitals NHS Foundation Trust, Blackpool, UK; Hull York Medical School, University of Hull, Hull, UK; Ninewells Hospital and Medical School, Dundee, UK; University Hospitals Coventry and Warwickshire NHS Trust, Coventry, UK; Chesterfield Royal Hospital NHS Foundation Trust, Chesterfield, UK; NHS Fife, Kirkcaldy, UK; Imperial College Healthcare NHS Trust, London, UK; Princess of Wales Hospital, Bridgend, UK; School of Cardiovascular and Metabolic Health, University of Glasgow, 126 University Place, Glasgow, Lanarkshire, G12 8TA, UK; University Hospital Monklands, Airdrie, UK; School of Cardiovascular and Metabolic Health, University of Glasgow, 126 University Place, Glasgow, Lanarkshire, G12 8TA, UK; Robertson Centre for Biostatistics, University of Glasgow, Glasgow, UK; Robertson Centre for Biostatistics, University of Glasgow, Glasgow, UK; Robertson Centre for Biostatistics, University of Glasgow, Glasgow, UK; Robertson Centre for Biostatistics, University of Glasgow, Glasgow, UK; Department of Cardiology, Portsmouth Hospitals University NHS Trust, Portsmouth, UK; Faculty of Science and Health, University of Portsmouth, Portsmouth, UK; College of Medical, Veterinary and Life Sciences, University of Glasgow, Glasgow, UK

**Keywords:** Heart failure, Anaemia, Iron deficiency, Ferric derisomaltose, Randomized clinical trial, Transferrin saturation, Ferritin, Quality of life, Morbidity, Mortality

## Abstract

**Background and Aims:**

What is the relationship between blood tests for iron deficiency, including anaemia, and the response to intravenous iron in patients with heart failure?

**Methods:**

In the IRONMAN trial, 1137 patients with heart failure, ejection fraction ≤ 45%, and either serum ferritin < 100 µg/L or transferrin saturation (TSAT) < 20% were randomized to intravenous ferric derisomaltose (FDI) or usual care. Relationships were investigated between baseline anaemia severity, ferritin and TSAT, to changes in haemoglobin from baseline to 4 months, Minnesota Living with Heart Failure (MLwHF) score and 6-minute walk distance achieved at 4 months, and clinical events, including heart failure hospitalization (recurrent) or cardiovascular death.

**Results:**

The rise in haemoglobin after administering FDI, adjusted for usual care, was greater for lower baseline TSAT (*P*_interaction_ < .0001) and ferritin (*P*_interaction_ = .028) and more severe anaemia (*P*_interaction_ = .014). MLwHF scores at 4 months were somewhat lower (better) with FDI for more anaemic patients (overall *P*_interaction_ = .14; physical *P*_interaction_ = .085; emotional *P*_interaction_ = .043) but were not related to baseline TSAT or ferritin. Blood tests did not predict difference in achieved walking distance for those randomized to FDI compared to control. The absence of anaemia or a TSAT ≥ 20% was associated with lower event rates and little evidence of benefit from FDI. More severe anaemia or TSAT < 20%, especially when ferritin was ≥100 µg/L, was associated with higher event rates and greater absolute reductions in events with FDI, albeit not statistically significant.

**Conclusions:**

This hypothesis-generating analysis suggests that anaemia or TSAT < 20% with ferritin > 100 µg/L might identify patients with heart failure who obtain greater benefit from intravenous iron. This interpretation requires confirmation.

## Structured Graphical Abstract

**Structured Graphical Abstract:**
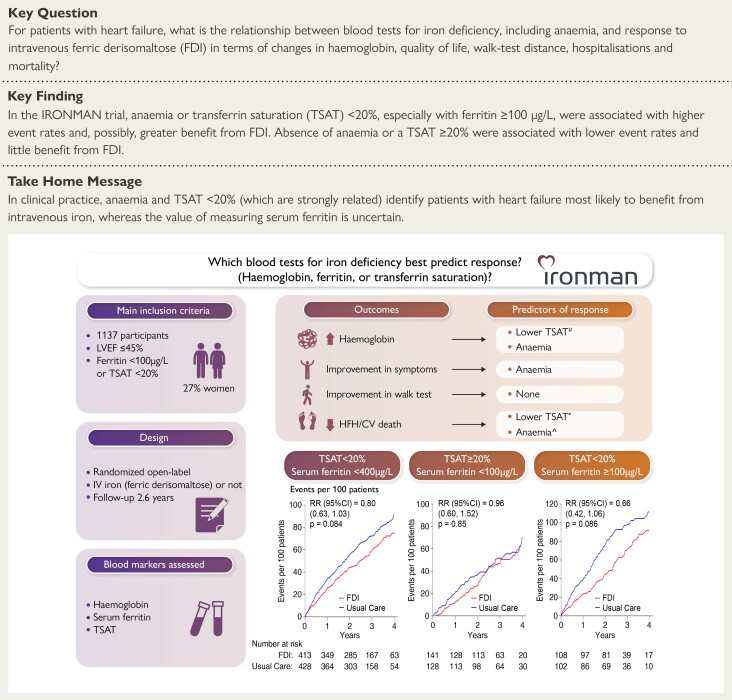
The IRONMAN trial main inclusion criteria, design, and blood markers of iron deficiency, including haemoglobin, transferrin saturation (TSAT), and serum ferritin, used to predict response to intravenous ferric derisomaltose (FDI). Markers of response including increase in haemoglobin, symptoms (Minnesota Living with Heart Failure questionnaire) and 6-minute walk test distance at 4 months and incidence of clinical events, including heart failure hospitalization (HFH) and cardiovascular (CV) death, for the duration of the trial with or without COVID-sensitivity analyses. CI, confidence interval; LVEF, left ventricular ejection fraction; RR, rate ratio. #Serum ferritin < 30 µg/L predicted a marked increase in haemoglobin only when associated with a TSAT < 20%. *Patients with a TSAT < 20% but serum ferritin ≥ 100 µg/L obtained the greatest benefit in terms of improvement in the primary endpoint. ^The benefit of FDI was similar in relative terms for patients with and without anaemia but because those with anaemia were at greater risk, they obtained greater absolute benefit.

**See the editorial comment for this article ‘Intravenous iron, only for those in need’, by P. van der Meer and N.G. Beverborg, https://doi.org10.1093/eurheartj/ehae093.**

## Introduction

For patients with heart failure and a reduced left ventricular ejection fraction (LVEF) who are thought to have iron deficiency, intravenous (i.v.) administration of iron increases haemoglobin, improves symptoms, reduces hospitalizations for heart failure, and might also reduce cardiovascular mortality.^1^ However, uncertainty exists about which blood markers best reflect iron deficiency and the response to i.v. iron.^2^ Administration of i.v. iron to patients who are not iron deficient may not be effective, which will dilute the benefit observed in clinical trials leading to underestimation of benefit and, potentially, a neutral outcome.

Unfortunately, there is no generally agreed definition of iron deficiency. The World Health Organization (WHO) defines it as a serum ferritin < 15 µg/L or, in the presence of inflammatory disease, <70 µg/L.^3^ Although many patients with heart failure do have activation of inflammatory pathways, this is usually low-grade.^4^ Many clinical laboratories define a low serum ferritin as ≤30 µg/L. There is greater consensus for transferrin saturation (TSAT), with values < 20% generally accepted as evidence of iron deficiency. Serum concentrations of iron^2,5^ or soluble transferrin receptors^6,7^ might be better markers of iron deficiency, but neither has been measured routinely in substantial randomized trials of i.v. iron.

Most trials of i.v. iron for heart failure have adopted criteria for iron deficiency similar to those for the FAIR-HF trial, namely a serum ferritin < 100 µg/L regardless of TSAT or 100–299 µg/L if TSAT is <20%.^1^ The basis for this definition is unclear. For patients with heart failure, a low serum ferritin appears inferior to a low TSAT as a marker of low iron stores or defective iron utilization in bone marrow.^8^ Also, for patients with chronic cardiovascular disease, a serum ferritin ≤ 30 µg/L is associated with a good prognosis, but a low TSAT with worse outcomes.^9,10^ The reasons for this paradox are uncertain. Low serum ferritin might reflect better cell health and membrane integrity, due to an absence of inflammation or other stressors, preventing leakage of ferritin rather than indicating iron deficiency, whereas a low TSAT may indicate low iron availability, regardless of whether this is due to absolute iron deficiency or sequestration in the reticulo-endothelial system.

Ultimately, the therapeutic response to iron supplements is the best guide to the clinical need for treatment with iron.^5,11^ However, several measures of response might be considered, including increases in haemoglobin, improvements in symptoms and exercise capacity, or reductions in hospitalization for heart failure or mortality. Accordingly, we now explore the relationship between baseline anaemia severity, serum ferritin, and TSAT, and the clinical response to i.v. iron in the IRONMAN trial.^12^

## Methods

IRONMAN was an investigator-initiated, randomized, open-label, blinded-endpoint, event-driven trial comparing i.v. ferric derisomaltose (FDI) or usual care, conducted in the UK.^12^ The trial was funded by the British Heart Foundation (grant award CS/15/1/31175). Pharmacosmos provided supplies of FDI and supported the trial with an additional unrestricted grant. The trial protocol and amendments were approved by a national ethics committee and other regulatory authorities.^12^ The trial design and main results have been published.^12,13^

Adults with new or established symptomatic heart failure and LVEF ≤ 45% were invited to participate. Following written informed consent, patients were screened for additional inclusion and exclusion criteria, with optional consent for record linkage to national databases for hospital admissions and deaths. To be included, haemoglobin had to be ≥9 g/dL for all participants, and ≤13 g/dL for women and ≤14 g/dL for men. Serum ferritin had to be <100 μg/L or TSAT < 20%. Patients with a serum ferritin > 400 μg/L were excluded based on the belief at the time of the trial design that this might reflect iron overload. Patients were also required either to have a current or recent (within 6 months) admission to hospital due to heart failure or to have raised plasma concentrations of natriuretic peptides.

Patients were reviewed 4 weeks after randomization and every 4 months thereafter until trial completion. Haemoglobin was measured at each trial visit in both groups but, in order not to encourage use of i.v. iron in those randomized to usual care, ferritin and TSAT were recorded only in those assigned to receive iron. For those assigned to FDI, investigators were asked to give further doses at trial visits if ferritin was <100 μg/L or, provided ferritin was ≤400 μg/L, a TSAT < 25%. Although patients assigned to usual care were not supposed to receive i.v. iron, they were permitted to have oral iron, although this was not actively encouraged.

The primary endpoint was hospitalization for heart failure or cardiovascular death analysed using a recurrent events analysis. A clinical event committee adjudicated, blind to treatment allocation, all deaths and unplanned hospital admissions. The Minnesota Living with Heart Failure (MLwHF) questionnaire^14^ and 6-minute walk distance were recorded at 4 months and 20 months.

Analysis of the primary outcome by anaemia severity, serum ferritin, and TSAT were individually pre-specified in the protocol but the interaction of these variables and investigation of effects on secondary outcomes were not.

For this analysis, the effects of FDI compared to control on the following outcomes were considered as potential markers of response:

1. Change in haemoglobin between baseline and 4 months;
2. MLwHF score, overall and its physical and emotional domains measured at 4 months. Note that this was achieved scores rather than change in status from baseline due to the variable context (inpatient vs. outpatient) of recruitment;
3. 6-Minute walk distance measured at 4 months. Note that this was also achieved distance rather than change in status from baseline;
4. The primary endpoint, (i) overall and (ii) censored on 30 September 2020, six months after the first COVID lockdown which, in the UK, effectively prevented further research visits and therefore further assessment and correction of iron deficiency; and
5. Cardiovascular and all-cause mortality, (i) overall and (ii) censored on 30 September 2020.

These outcomes were analysed with respect to:

1. Severity of anaemia (none, mild, or moderate), with mild anaemia being defined as 0–1 g/dL below and moderate anaemia as >1 g/dL below the WHO definition of anaemia (<12 g/dL for women; <13 g/dL for men). Note that, for men, this classification is slightly different from the primary statistical analysis plan that defined mild anaemia in men as 11.0–12.9 g/dL rather than 12.0–12.9 g/dL;
2. Serum ferritin (≤30 µg/L, 30–100 µg/L, and >100 µg/L; note that patients with serum ferritin > 100 µg/L were required to have a TSAT < 20%); and
3. TSAT (<10%, 10%–15%, 15–<20%, and ≥20%; note that patients with a TSAT ≥ 20% were required to have a serum ferritin < 100 µg/L).

### Statistical analysis

Variables that are continuously distributed at baseline are reported as median with first and third quartiles. Categorical variables are summarized as counts and percentages. Differences in baseline characteristics across subgroups are based on analysis of variance, Kruskal–Wallis tests, or χ^2^ tests as appropriate. Comparisons of outcomes between treatment arms within subgroup categories are based on two-sample *t*-tests for independent samples with corresponding 95% confidence intervals (CIs). Comparisons of differences of outcomes between treatment arms across subgroup categories are based on tests of interaction in analysis of variance. Recurrent event outcome comparisons between treatment arms are analysed using the method of Lin, Wei, Ying, and Yang,^15^ with the treatment effect expressed as a rate ratio and 95% CI. Comparisons of rate ratios across subgroup categories are analysed in corresponding models testing the interaction of treatment effect with patient classification according to anaemia severity, ferritin, TSAT, and combinations of ferritin and TSAT. Graphical presentations of cumulative events by subgroup category are based on the method of Ghosh and Lin,^16^ adjusting for the competing risk of deaths not included in the outcome. Time to first event outcomes is analysed in a similar manner using Cox proportional hazard models with treatment effects estimated as hazard ratios and 95% CI.

All *P*-values quoted are two-sided. The data were analysed using SAS version 9.4, R version 3.6.1 and Minitab version 20.3.

## Results

At baseline, measurements of haemoglobin, serum ferritin, and TSAT were available for 1137 (100%), 1135 (>99%), and 1111 (98%) patients. At 4 months, the increase in haemoglobin was greater for patients randomized to FDI compared to usual care (mean difference 0.6 [95% CI: 0.5 to 0.8] g/dL; *P* < .001) and patients assigned to FDI had a better MLwHFQ score (mean difference −3 [95% CI: −7 to 0]; *P* = .05), which was driven mainly by a difference in the physical domain (mean difference −2 [95% CI: −3 to −1]; *P* = .0071). For 648 patients with available data, the 6-minute walk test distance at 4 months was similar for patients randomized to FDI or control (mean difference −2 [95% CI: −28 to +25] m; *P* = .90). The reduction in the primary composite endpoint was of borderline statistical significance (rate ratio 0.82 [95% CI: 0.66 to 1.02]; *P* = .070) with a similar result in a COVID-sensitivity analysis (rate ratio 0.76 (95% CI: 0.58–1.00); *P* = .047). Trends to reductions in cardiovascular and all-cause mortality for patients assigned to FDI were not significant.

### Anaemia

Overall, 771 (68%) patients were anaemic, of whom 348 were considered to have mild anaemia and 423 moderate anaemia (>1 g/dL below the WHO definition but ≥9 g/dL). Anaemic patients were slightly older, were more likely to be men, were more symptomatic, and were more likely to have features associated with an adverse prognosis, including diabetes, higher plasma concentrations of N-terminal pro-B-type natriuretic peptide (NT-proBNP), lower estimated glomerular filtration rate (eGFR), treatment with loop diuretics, and lower rates of treatment with renin-angiotensin system antagonists (*Table 1*). Patients with moderate anaemia were more likely to have a serum ferritin ≤ 30 µg/L, TSAT < 20%, and to be receiving oral iron supplements. Patients with moderate anaemia had poorer MLwHF scores at baseline.

**Table 1:** Baseline characteristics according to anaemia category

Anaemia	Moderate	Mild	None	*P*
Haemoglobin for women	9.0–10.9 g/dL	11.0–11.9 g/dL	12.0–13.0 g/dL	
Haemoglobin for men	9.0–11.9 g/dL	12.0–12.9 g/dL	13.0–14.0 g/dL	
Total number	423	348	366	
Age	75 [68, 81]	73 [67, 80]	72 [66, 78]	.00068
Women	71 (17%)	98 (28%)	131 (36%)	<.0001
Men	352 (83%)	250 (72%)	235 (64%)	
BMI (kg/m^2)^	28.0 [24.5, 31.6]	28.4 [25.0, 32.7]	28.7 [24.6, 33.1]	.24
Medical history				
Hypertension	239 (57%)	187 (54%)	186 (51%)	.28
Diabetes	216 (51%)	172 (49%)	133 (36%)	<.0001
Atrial fibrillation	221 (52%)	150 (43%)	163 (45%)	.021
Aetiology				
Ischaemic	253 (60%)	195 (56%)	199 (54%)	.55
Non-ischaemic	129 (31%)	114 (33%)	130 (36%)	
Unknown	41 (10%)	39 (11%)	37 (10%)	
Recruitment context				
Inpatient	90 (21%)	40 (12%)	34 (9%)	<.0001
Discharged <6 months	76 (18%)	63 (18%)	69 (19%)	
Outpatient	257 (61%)	245 (70%)	263 (72%)	
NYHA				
II	193 (46%)	214 (62%)	241 (66%)	<.0001
III/IV	230 (54%)	134 (38%)	125 (34%)	
Minnesota score (*n*)	412	341	361	
Overall	49 [29, 66]	42 [24, 58]	39 [19, 60]	<.0001
Physical	26 [16, 33]	23 [12, 31]	22 [12, 29]	.0003
Emotional	10 [4, 17]	8 [3, 15]	7 [2, 16]	.0033
6-Minute walk test (*n*)	231	204	226	
Distance (m)	235 [149, 322]	270 [180, 361]	300 [201, 370]	<.0001
Vital signs				
Heart rate (beats/min)	70 [60, 79]	70 [61, 81]	69 [60, 78]	.40
Systolic BP (mmHg)	117 [103, 130]	120 [108, 134]	120 [108, 133]	.011
Laboratory tests				
LVEF (%)	35 [26, 39]	31 [25, 36]	35 [25, 38]	.021
NT-proBNP (ng/L)	2424 [1142, 4329]	1562 [838, 3541]	1387 [741, 2603]	<.0001
eGFR (mL/min/1.73 m^2^)	47 [35, 62]	52 [38, 72]	56 [41, 72]	<.0001
Haemoglobin (g/dL)	10.9 [10.3, 11.5]	12.3 [11.8, 12.6]	13.1 [12.7, 13.6]	NA
Ferritin (µg/L)	50 [29, 94]	44 [28, 83]	54 [32, 83]	.071
Ferritin < 30 µg/L	112 (27%)	108 (31%)	77 (21%)	.01
TSAT (%)	12 [9, 18]	15 [11, 19]	17 [13, 22]	<.0001
TSAT < 20%	345 (84%)	258 (76%)	238 (66%)	<.0001
On oral iron	80 (19%)	56 (16%)	35 (10%)	.00098
Heart failure medication				
Loop diuretic	371 (88%)	274 (79%)	281 (77%)	.0001
ACEi, ARB, or ARNi	343 (81%)	310 (89%)	331 (90%)	.00016
Beta-blocker	367 (87%)	314 (90%)	328 (90%)	.26
MRA	222 (53%)	211 (61%)	199 (54%)	.065
Digoxin	49 (12%)	45 (13%)	41 (11%)	.75
Any hypoglycaemic agent	195 (46%)	157 (45%)	110 (30%)	<.0001
Insulin	89 (21%)	53 (15%)	39 (11%)	.00034
SGLT2 inhibitor	11 (3%)	10 (3%)	8 (2%)	.84
Device therapy				
ICD	57 (14%)	46 (13%)	60 (16%)	.40
PPCM	30 (7%)	15 (4%)	21 (6%)	
CRT-P	30 (7%)	25 (7%)	23 (6%)	
CRT-D	55 (13%)	62 (18%)	48 (13%)	

Data are number and per cent or median with first and third quartiles.

**Figure 1:**
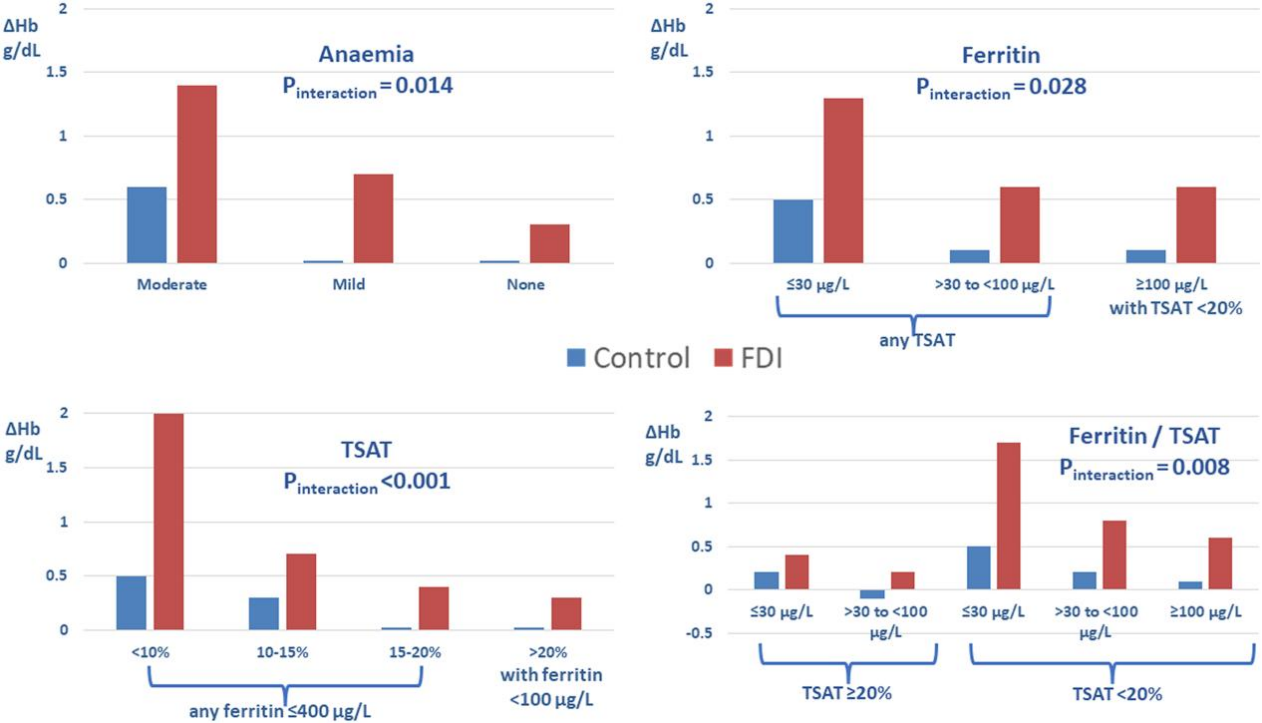
Change in haemoglobin (ΔHb) for patients randomized to usual care (control) or ferric derisomaltose (FDI) according to anaemia status, serum ferritin, and transferrin saturation (TSAT). Mean changes are shown. See *Tables 2, 3, 5,* and *6* for standard deviations of change and mean differences with 95% confidence intervals

Four months after administration of FDI, haemoglobin rose by a mean (SD) of 1.4 (1.5) g/dL, 0.7 (1.2) g/dL, and 0.3 (1.0) g/dL, respectively, for patients with moderate, mild, or no anaemia according to the WHO definition. An interaction between anaemia severity and the rise in haemoglobin with FDI was observed (*P*_interaction_ = .014) (*Table 2*, *Figure 1*).

**Table 2:** Effects of ferric derisomaltose compared to usual care for patients classified by severity of anaemia

Anaemia	Moderate		Mild		None		*P* for interaction
	Usual care	FDI	Usual care	FDI	Usual care	FDI	
	214	209	160	188	194	172	
Haemoglobin (g/dL)							
Value at 4 months	11.5 (1.5)	12.2 (1.5)	12.1 (1.0)	13.0 (1.2)	13.1 (1.2)	13.3 (1.1)	
Difference in change from baseline	0.8 (0.5 to 1.1)		0.8 (0.5 to 1.0)		0.3 (0.0 to 0.6)		.014
MLwHF score overall (*n*)	181	184	133	167	171	151	
Value at 4 months	47 (27)	39 (30)	38 (25)	35 (26)	36 (25)	36 (25)	
Difference at 4 months	−7 (−13 to −2)		−3 (−9 to + 3)		+1 (−5 to +6)		.14
MLwHF score physical							
Value at 4 months	23 (11)	19 (13)	20 (11)	17 (12)	18 (11)	18 (11)	
Difference at 4 months	−4 (−6 to −1)		−2 (−5 to +1)		+0 (−2 to +3)		.085
MLwHF score emotional							
Value at 4 months	11 (8)	9 (8)	8 (8)	8 (7)	8 (8)	8 (8)	
Difference at 4 months	−2 (−4 to −0)		0 (−1 to +2)		+1 (−1 to +2)		.043
6-Minute walk test (*n*)	87	91	63	82	77	84	
Distance at 4 months (m)	263 (119)	244 (132)	267 (129)	294 (136)	310 (142)	312 (117)	
Difference usual care vs. FDI	−19 (−57 to +18)		+27 (−17 to +71)		+2 (−38 to +43)		.28
Primary endpoint							
Rate per 100 patient-years	38.9	30.5	24.6	20.4	19.2	16.8	
Rate ratio	0.81 (0.59 to 1.12)		0.83 (0.54 to 1.28)		0.86 (0.58 to 1.29)		.95
Absolute difference in rate	**8.4 lower with FDI**		4.2 lower with FDI		2.4 lower with FDI		
Primary endpoint (COVID sensitivity)							
Rate per 100 patient-years	43.3	33.7	23.1	17.0	20.2	16.9	
Rate ratio	0.80 (0.55 to 1.18)		0.74 (0.42 to 1.30)		0.82 (0.50 to 1.34)		.91
Absolute difference in rate	**9.6 lower with FDI**		**6.1 lower with FDI**		3.3 lower with FDI		
CV mortality							
Percentage (%)	35.0	26.8	21.3	18.1	14.9	16.9	
Hazard ratio	0.80 (0.56 to 1.13)		0.80 (0.50 to 1.30)		1.13 (0.67 to 1.89)		.55
Absolute difference in percentages (%)	**8.2 lower with FDI**		3.2 lower with FDI		2.0 higher with FDI		
CV mortality (COVID sensitivity)							
Percentage (%)	24.0	21.2	13.0	6.9	9.7	9.1	
Hazard ratio	0.93 (0.61 to 1.42)		0.52 (0.25 to 1.08)		0.89 (0.45 to 1.76)		.33
Absolute difference in percentages (%)	2.8 lower with FDI		**6.1 lower with FDI**		0.6 higher with FDI		
All-cause mortality							
Percentage (%)	48.1	41.1	29.4	25.5	22.2	29.1	
Hazard ratio	0.91 (0.68 to 1.21)		0.80 (0.54 to 1.20)		1.29 (0.86 to 1.94)		.22
Absolute difference in percentages (%)	**7.0 lower with FDI**		3.9 lower with FDI		**6.9 higher with FDI**		
All-cause mortality (COVID sensitivity)							
Percentage (%)	31.4	30.7	18.5	12.1	12.9	14.6	
Hazard ratio	1.06 (0.74 to 1.51)		0.62 (0.35 to 1.10)		1.07 (0.61 to 1.88)		.24
Absolute difference in percentages (%)	0.7 lower with FDI		**6.4 lower with FDI**		1.7 higher with FDI		

Substantial differences in rates are highlighted in bold. MLwHF, Minnesota Living with Heart Failure.

**Figure 2:**
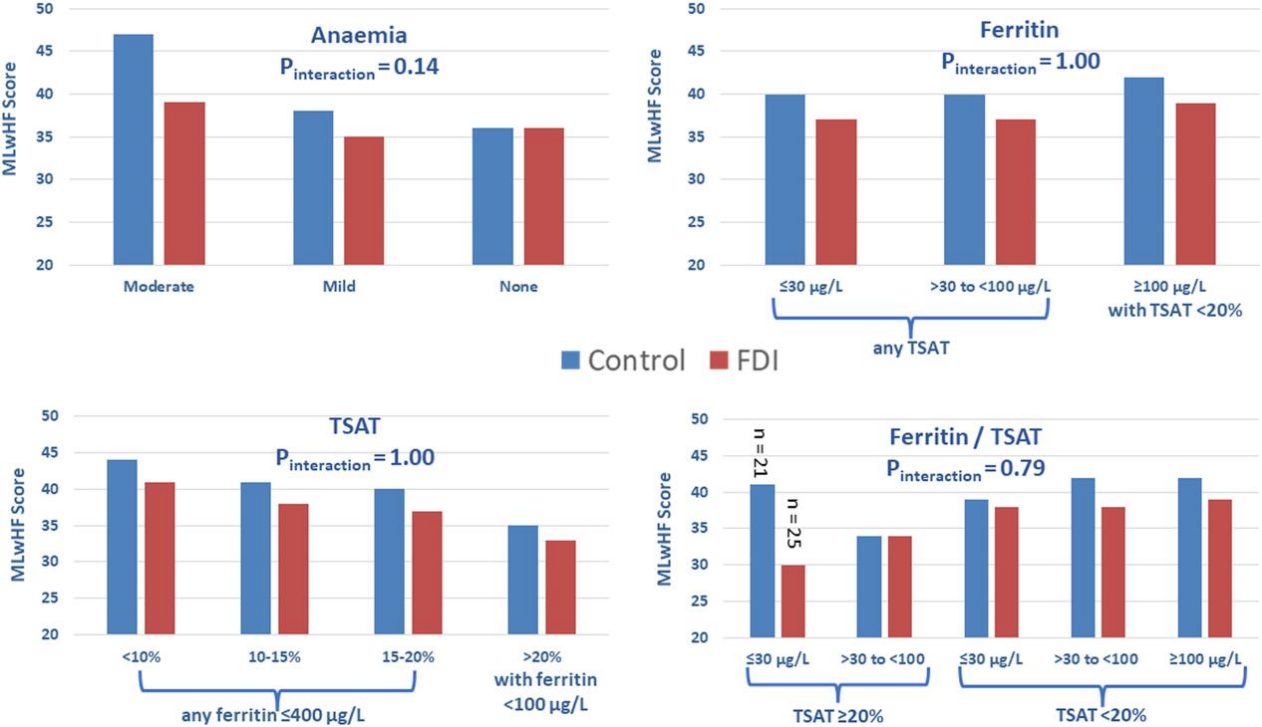
Differences in Minnesota Living with Heart Failure questionnaire score (lower is better) at 4 months for patients randomized to usual care (control) or ferric derisomaltose (FDI) according to anaemia status, serum, ferritin and transferrin saturation (TSAT). Mean values are shown. See *Tables 2, 3, 5,* and *6* for standard deviations of change and mean differences with 95% confidence intervals

For those with moderate anaemia at baseline, MLwHF total score at 4 months favoured those assigned to i.v. FDI compared to control (mean difference −7 [95% CI: −13 to −2]) but little difference was observed for those with mild anaemia (mean difference −3 [95% CI: −9 to +3]) or without anaemia (mean difference +1 [95% CI: −5 to +6]). However, tests for interaction between anaemia severity and the effects of treatment on MLwHF were not significant (*P*_interaction_ = .14) (*Table 2*, *Figure 2*). Similar patterns were observed for both the physical (*P*_interaction_ = .085) and emotional (*P*_interaction_ = .043) domains. Administration of FDI was not associated with a greater walk distance for any anaemia category (*Table 2*).

**Figure 3:**
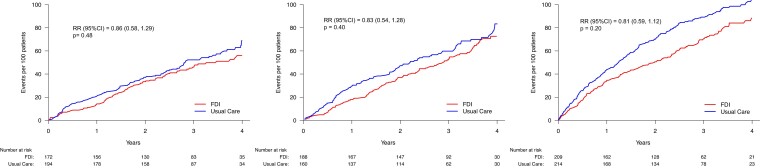
Primary endpoint (recurrent events) according to anaemia classification (none, mild, or moderate). No statistically significant interaction was observed between anaemia severity and treatment effect (*P*_interaction_ = .95). FDI, ferric derisomaltose; RR, rate ratio. See *Table 1* for definition of anaemia classes and *Table 2* for event rates

Compared to patients with mild or no anaemia at baseline, those with moderate anaemia assigned to usual care had higher rates for the primary composite endpoint. FDI exerted a greater absolute reduction in event rate for patients with moderate anaemia, but the rate ratio was similar regardless of anaemia severity (*P*_interaction_ = .95) (*Table 2*, *Figure 3*). Similar patterns were noted in COVID-sensitivity analyses and for cardiovascular and all-cause mortality (*Table 2*).

### Serum ferritin

Only 82 patients (7%) had a serum ferritin < 15 µg/L. Serum ferritin was ≤30 µg/L in 297 patients (26%), 30 µg/L to <100 µg/L in 627 (55%), and ≥100 µg/L in 210 (19%). Patients with a ferritin ≤ 30 µg/L were more likely to be women and generally had a more favourable prognostic profile, mostly being enrolled as outpatients in New York Heart Association (NYHA) functional class II, with lower NT-proBNP, higher eGFR, and less often treated with loop diuretics (see Supplementary data online, *Table S1A*). Patients with a serum ferritin ≤ 30 µg/L had only a slightly higher prevalence of anaemia but lower values for TSAT. Conversely, patients with a serum ferritin ≥ 100 µg/L (and consequently TSAT < 20%) had a worse prognostic profile in most respects than patients with a ferritin < 100 µg/L.

By 4 months, the increase in haemoglobin was greater in patients with a serum ferritin ≤ 30 µg/L randomized to FDI (*P*_interaction_ = .028) (*Table 3*, *Figure 1*). There was no difference in the effect of FDI on MLwHF by serum ferritin category (*P*_interaction_ = 1.0), with similar findings for physical (*P*_interaction_ = .85) or emotional (*P*_interaction_ = .90) domains (*Table 3*, *Figure 2*). For patients with a serum ferritin ≥ 100 µg/L (and therefore TSAT < 20%), walk distance tended to be greater for patients randomized to FDI (mean increase 40 (95% CI −12 to +93)) but no effect was observed for other groups (*Table 3*).

**Table 3:** Effects of ferric derisomaltose compared to usual care for patients classified by serum ferritin concentration

Serum ferritin concentration	≤30 µg/L		>30 to <100 µg/L		≥100 μg/L*		*P* for interaction
	Usual Care	FDI	Usual Care	FDI	Usual Care	FDI	
	146	151	317	310	102	108	
Haemoglobin (g/dL)							
Value at 4 months	12.5 (1.5)	13.4 (1.3)	12.2 (1.4)	12.7 (1.3)	12.0 (1.4)	12.3 (1.4)	
Difference in change from baseline	1.0 (0.6 to 1.3)		0.5 (0.3 to 0.7)		0.5 (0.1 to 0.8)		.028
MLwHF score overall (*n*)	129		263		90	91	
Value at 4 months	40 (25)	37 (27)	40 (27)	37 (27)	42 (27)	39 (28)	
Difference at 4 months	−3 (−10 to +3)		−3 (−8 to +1)		−3 (−11 to +5)		1.0
MLwHF score physical							
Value at 4 months	20 (11)	17 (11)	20 (12)	18 (12)	21 (12)	19 (12)	
Difference at 4 months	−3 (−6 to 0)		−2 (−4 to +0)		−1 (−5 to +2)		.77
MLwHF score emotional							
Value at 4 months	9 (8)	9 (8)	9 (8)	8 (8)	9 (7)	9 (8)	
Difference at 4 months	+0 (−2 to +2)		−1 (−2 to +1)		−1 (−3 to +2)		.67
6-Minute walk test (*n*)	65	64	124	149	38	44	
Distance at 4 months (m)	291 (120)	287 (140)	287 (139)	281 (130)	240 (116)	280 (122)	
Difference usual care vs. FDI	−4 (−50 to +41)		−6 (−38 to +26)		+40 (−12 to +93)		.36
Primary endpoint							
Rate per 100 patient-years	17.6%	13.8%	28.4%	25.2%	39.7%	26.9%	
Rate ratio	0.79 (0.47 to 1.31)		0.90 (0.68 to 1.18)		0.66 (0.42 to 1.06)		.57
Absolute difference in rate	3.8% lower with FDI		3.2% lower with FDI		**12.8% lower with FDI**		
Primary endpoint (COVID sensitivity)							
Rate per 100 patient-years	16.4%	12.6%	30.8%	26.4%	43.7%	24.3%	
Rate ratio	0.76 (0.41 to 1.43)		0.87 (0.62 to 1.23)		0.53 (0.29 to 0.98)		.39
Absolute difference in rate	3.8% lower with FDI		4.4% lower with FDI		**19.4% lower with FDI**		
CV mortality							
Percentage (%)	13.7%	13.9%	27.8%	24.5%	27.5%	20.4%	
Hazard ratio	1.08 (0.59 to 2.00)		0.87 (0.64 to 1.19)		0.70 (0.40 to 1.22)		.46
Absolute difference in percentages (%)	0.2% higher with FDI		3.3% lower with FDI		**7.1% lower with FDI**		
CV mortality (COVID sensitivity)							
Percentage (%)	5.8%	9.2%	19.4%	15.1%	20.0%	11.0%	
Hazard ratio	1.66 (0.69 to 4.01)		0.76 (0.51 to 1.12)		0.56 (0.26 to 1.18)		.15
Absolute difference in percentages (%)	3.4% higher with FDI		4.3% lower with FDI		**9.0% lower with FDI**		
All-cause mortality							
Percentage (%)	24.7%	27.2%	36.0%	36.5%	40.2%	27.8%	
Hazard ratio	1.17 (0.75 to 1.83)		1.01 (0.78 to 1.31)		0.65 (0.41 to 1.05)		.16
Absolute difference in percentages (%)	2.5% higher with FDI		0.5% higher with FDI		**12.4% lower with FDI**		
All-cause mortality (COVID sensitivity)							
Percentage (%)	12.9%	14.6%	24.1%	22.1%	25.3%	15.0%	
Hazard ratio	1.07 (0.61 to 1.88)		0.90 (0.64 to 1.26)		0.59 (0.31 to 1.13)		.14
Absolute difference in percentages (%)	1.7% higher with FDI		2.0% lower with FDI		**10.3% lower with FDI**		

Substantial differences in rates are highlighted in bold. MLwHF, Minnesota Living with Heart Failure.

*TSAT <20% required if ferritin ≥100 μg/L. One patient assigned to usual care did not have a measurement of TSAT but is included here.

**Figure 4:**
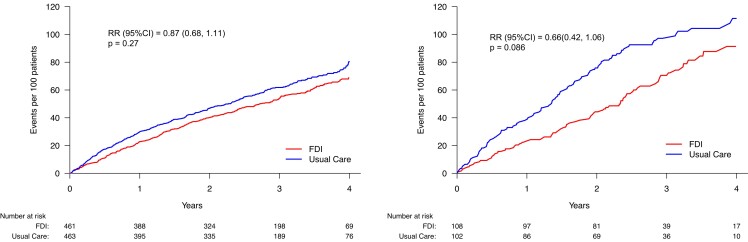
Primary endpoint (recurrent events) according to serum ferritin < 100 or ≥100 µg/L (with TSAT < 20%). No statistically significant interaction was observed between serum ferritin classification and treatment effect. FDI, ferric derisomaltose; RR, rate ratio. See *Table 3* for event rates

In both treatment arms, patients with a serum ferritin ≤ 30 µg/L had the lowest rate for the primary endpoint, with slightly lower rates in those assigned to FDI, compared to usual care. Those with a serum ferritin ≥ 100 µg/L (and consequently a TSAT < 20%) had the highest rate for the primary endpoint and the greatest absolute reduction in event rate with FDI. However, the interaction between assigned treatment and ferritin category was not statistically significant (*P*_interaction_ = .57) (*Table 3*, *Figure 4*). Similar patterns were noted in COVID-sensitivity analyses and for cardiovascular and all-cause mortality (*Table 3*).

### Transferrin saturation

TSAT was <20% in 841 patients (76%), of whom almost one-third (272 patients) had values ≤ 10%. Age and sex did not differ by TSAT category. Patients with a low TSAT were more symptomatic, had a lower LVEF, higher NT-proBNP, and were more likely to receive loop diuretics, although eGFR was similar across categories (*Table 4*). Patients with a low TSAT were also more likely to be anaemic and had lower serum ferritin. Patients with TSAT ≥ 20% who, by protocol were required to have a serum ferritin < 100 µg/L, had a more favourable prognostic profile than those with a TSAT < 20%.

**Table 4:** Baseline characteristics according to transferrin saturation category

TSAT	≤10%	>10% to ≤15%	>15% to <20%	>20%	*P*
Total number	272	298	271	269	
Age (years)	73 [64, 79]	73 [67, 79]	74 [67, 79]	74 [68, 80]	.19
Women	80 (29%)	85 (29%)	66 (24%)	61 (23%)	.22
Men	192 (71%)	213 (72%)	205 (76%)	208 (77%)	
BMI (kg/m^2^)	28.7 [24.8, 33.3]	28.7 [25.1, 32.2]	28.4 [24.5, 32.9]	27.4 [24.5, 31.3]	.094
Medical history					
Hypertension	160 (59%)	168 (56%)	137 (51%)	130 (48%)	.048
Diabetes	130 (48%)	145 (49%)	118 (44%)	113 (42%)	.32
Atrial fibrillation	138 (51%)	137 (46%)	126 (47%)	116 (43%)	.36
Aetiology					
Ischaemic	138 (51%)	175 (59%)	158 (58%)	163 (61%)	.24
Non-ischaemic	104 (38%)	89 (30%)	89 (33%)	79 (29%)	
Unknown	30 (11%)	34 (11%)	24 (9%)	27 (10%)	
Recruitment context					
Inpatient	75 (28%)	33 (11%)	26 (10%)	14 (5%)	<.0001
Discharged <6 months	50 (18%)	60 (20%)	46 (17%)	50 (19%)	
Outpatient	147 (54%)	205 (69%)	199 (73%)	205 (76%)	
NYHA					
II	144 (53%)	163 (55%)	148 (55%)	183 (68%)	.00073
III/IV	128 (47%)	135 (45%)	123 (45%)	86 (32%)	
Minnesota (*n*)	264	292	266	268	
Overall	51 [31, 68]	43 [26, 61]	41 [22, 66]	36 [19, 58]	<.0001
Physical	26 [16, 33]	24 [15, 31]	23 [12, 30]	20 [11, 29]	.00018
Emotional	10 [4, 18]	8 [3, 15]	8 [2, 15]	7 [7, 15]	.035
6-Minute walk test (*n*)	137	171	165	179	
Distance (m)	240 [142, 335]	288 [174, 351]	259 [173, 330]	300 [204, 365]	.0053
Vital signs					
Heart rate (beats/min)	73 (63, 84)	69 (60, 78)	69 (61, 78)	65 (60, 75)	<.0001
Systolic BP (mmHg)	116 (105, 128)	118 (105, 133)	122 (109, 134)	119 (105, 132)	.12
Laboratory tests					
LVEF	33 (24, 36)	35 (26, 40)	31 (25, 36)	35 (29, 39)	.0013
NT-proBNP	2112 (967, 4096)	1905 (984, 3818)	1717 (851, 3952)	1373 (823, 2694)	.039
eGFR (mL/min/1.73 m^2^)	53 (37, 74)	51 (38, 65)	49 (38, 68)	53 (40, 70)	.36
Haemoglobin (g/dL)	11.4 (10.6, 12.2)	12.1 (11.1, 12.8)	12.3 (11.5, 12.9)	12.6 (11.8, 13.1)	<.0001
No anaemia	46 (17%)	89 (30%)	103 (38%)	123 (46%)	<.0001
Mild anaemia	72 (27%)	99 (33%)	87 (32%)	82 (31%)	
Moderate anaemia	154 (57%)	110 (37%)	81 (30%)	64 (24%)	
Ferritin (µg/L)	32 (17, 54)	55 (29, 96)	76 (39, 137)	53 (35, 76)	<.0001
Ferritin ≤ 30 µg/L	124 (46%)	79 (27%)	39 (14%)	46 (17%)	<.0001
On oral iron	31 (11%)	50 (17%)	46 (17%)	40 (15%)	.23
Heart failure medication					
Loop diuretic	226 (83%)	247 (83%)	226 (83%)	204 (76%)	.068
ACEi, ARB, or ARNi	221 (81%)	257 (86%)	236 (87%)	251 (93%)	.00056
Beta-blocker	238 (88%)	265 (89%)	243 (90%)	239 (89%)	.88
MRA	150 (55%)	157 (53%)	149 (55%)	164 (61%)	.24
Digoxin	36 (13%)	40 (13%)	27 (10%)	28 (10%)	.45
Any hypoglycaemic agent	122 (45%)	128 (43%)	104 (38%)	96 (36%)	.11
Insulin	48 (18%)	51 (17%)	53 (19%)	27 (10%)	.019
SGLT2 inhibitors	15 (6%)	5 (2%)	4 (2%)	4 (2%)	.0043
Device therapy—no. (%)					
ICD	43 (16%)	47 916%)	37 (14%)	34 (13%)	.51
PPM	18 (7%)	21 (7%)	14 (5%)	10 (4%)	
CRT-P	21 (8%)	14 (5%)	24 (9%)	18 (7%)	
CRT-D	33 (12%)	41 (59%)	42 (16%)	45 (17%)	

Data are number and per cent or median with first and third quartiles.

The increase in haemoglobin in response to FDI was greatest for patients with a TSAT < 10% (*P*_interaction_ < .001), with a progressively smaller response for higher values of TSAT (*Table 5*, *Figure 1*). There was little difference in the improvement in MLwHF scores across TSAT categories, overall (*P*_interaction_ = 1.0) or for physical (*P*_interaction_ = .77) or emotional (*P*_interaction_ = .67) domains (*Table 5*, *Figure 2*). No differences between FDI and control in walk distance were observed according to TSAT (*Table 5*).

**Table 5:** Effects of ferric derisomaltose compared to usual care for patients classified by transferrin saturation

TSAT	≤10		>10 to ≤15		>15 to <20		≥ 20%*		*P* for interaction
	Usual care	FDI	Usual care	FDI	Usual care	FDI	Usual care	FDI	
	142	130	142	156	144	127	128	141	
Haemoglobin (g/dL)									
Value at 4 months	12.1 (1.6)	13.4 (1.4)	12.3 (1.3)	12.7 (1.4)	12.1 (1.4)	12.5 (1.3)	12.4 (1.4)	12.7 (1.1)	
Difference in change from baseline	1.4 (1.0 to 1.8)		0.4 (0.1 to 0.7)		0.5 (0.2 to 0.8)		0.3 (0.1 to 0.6)		<.001
MLwHF score overall (*n*)									
Value at 4 months	44 (29)	41 (30)	41 (26)	38 (28)	40 (26)	37 (27)	35 (25)	33 (24)	
Difference at 4 months	−3 (−11 to +5)		−3 (−10 to +4)		−3 (−10 to +4)		−2 (−8 to +4)		1.0
MLwHF score physical									
Value at 4 months	22 (12)	19 (13)	20 (11)	18 (12)	20 (11)	18 (12)	18 (11)	17 (11)	
Difference at 4 months	−3 (−6 to +0)		−2 (−5 to +1)		−2 (−5 to +1)		−1 (−4 to +2)		.85
MLwHF score emotional									
Value at 4 months	9 (8)	10 (9)	9 (8)	8 (8)	9 (7)	8 (8)	8 (8)	7 (7)	
Difference at 4 months	+0 (−2 to +3)		−1 (−2 to +2)		−1 (−3 to +2)		−1 (−3 to +1)		.90
6-Minute walk test (*n*)	51	50	52	65	59	58	61	79	
Distance at 4 months (m)	256 (125)	265 (116)	283 (145)	295 (142)	262 (124)	264 (127)	324 (121)	297 (134)	
Difference usual care vs. FDI	+9 (−39 to +57)		+12 (−41 to +65)		+1 (−45 to +47)		−27 [−71 to +16]		.61
Primary endpoint									
Rate per 100 patient-years	32.1	25.4	24.1	24.3	32.6	21.4	20.3	17.8	
Rate ratio	0.80 (0.53 to 1.21)		0.99 (0.64 to 1.54)		0.65 (0.42 to 1.01)		0.96 (0.60 to 1.52)		.58
Absolute difference in rate	**6.7 lower with FDI**		0.2 higher with FDI		**11.2 lower with FDI**		2.5 lower with FDI		
Primary endpoint (COVID sensitivity)									
Rate per 100 patient-years	30.1	29.1	28.5	20.7	36.5	20.3	18.8	17.4	
Rate ratio	0.95 (0.58 to 1.55)		0.72 (0.42 to 1.22)		0.55 (0.31 to 0.98)		0.96 (0.55 to 1.68)		.50
Absolute difference in rate	1.0 lower with FDI		**7.8 lower with FDI**		**16.2 lower with FDI**		1.4 lower with FDI		
CV mortality									
Percentage (%)	26.8	23.8	21.8	21.8	27.8	18.9	18.8	17.7	
Hazard ratio	0.90 (0.56 to 1.45)		1.05 (0.64 to 1.70)		0.63 (0.38 to 1.05)		1.03 (0.59 to 1.82)		.46
Absolute difference in percentages (%)	3.0 lower with FDI		No difference		**8.9 lower with FDI**		1.0 lower with FDI		
CV mortality (COVID sensitivity)									
Percentage (%)	14.4	15.7	14.6	11.7	20.4	9.2	11.9	11.9	
Hazard ratio	1.06 (0.55 to 2.01)		0.80 (0.42 to 1.53)		0.42 (0.21 to 0.84)		1.12 (0.54 to 2.29)		.18
Absolute difference in percentages (%)	1.3 higher with FDI		2.9 lower with FDI		**11.2 lower with FDI**		No change		
All-cause mortality									
Percentage (%)	40.8	38.5	29.6	32.1	36.8	27.6	25.8	30.5	
Hazard ratio	0.96 (0.66 to 1.40)		1.13 (0.75 to 1.70)		0.69 (0.45 to 1.06)		1.30 (0.82 to 2.05)		.21
Absolute difference in percentages (%)	2.3 lower with FDI		2.5 higher with FDI		**9.2 lower with FDI**		4.7 higher with FDI		
All-cause mortality (COVID sensitivity)									
Percentage (%)	24.2	24.3	19.0	16.6	24.8	16.0	14.4	20.0	
Hazard ratio	0.99 (0.60 to 1.64)		0.88 (0.50 to 1.53)		0.58 (0.33 to 1.02)		1.56 (0.85 to 2.87)		.15
Absolute difference in percentages (%)	0.1 higher with FDI		2.4 lower with FDI		**8.8 lower with FDI**		**5.6 higher with FDI**		

Substantial differences in rates are highlighted in bold. MLwHF, Minnesota Living with Heart Failure.

*Ferritin <100 μg/L required if TSAT ≥20%.

**Figure 5:**
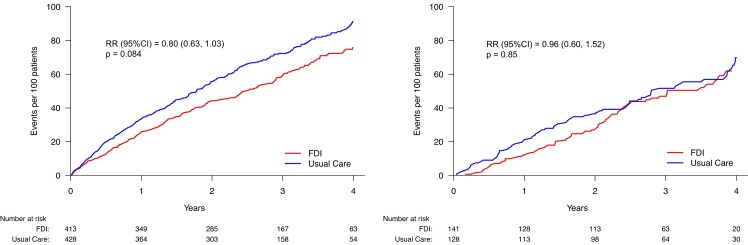
Primary endpoint (recurrent events) according to transferrin saturation (TSAT) < 20% or ≥20% (with serum ferritin < 100 µg/L). No statistically significant interaction was observed between TSAT classification and treatment effect. FDI, ferric derisomaltose; RR, rate ratio. See *Table 5* for event rates

Compared to patients with TSAT ≥ 20%, those with TSAT < 20% had a higher rate for the primary endpoint and a greater reduction with FDI, but this effect was not consistent across categories of TSAT below 20%. This may reflect the play of chance due to small numbers of patients and events in each subgroup. In COVID-sensitivity analyses, results were unstable for patients with a TSAT < 10% or 10%–15%. Patients with TSAT ≥ 20% (and consequently serum ferritin < 100 µg/L) were at lower risk of events and appeared to derive little benefit from FDI, although a trend was observed in the first 18 months (*Figure 5*). However, the interaction between TSAT category and assigned treatment was not statistically significant (*P*_interaction_ = .58) (*Table 5*). Similar patterns were noted in COVID-sensitivity analyses and for cardiovascular and all-cause mortality (*Table 5*).

In an analysis including only the 841 patients with a TSAT < 20%, the reduction in the primary composite endpoint (*Figure 5*) was again of borderline statistical significance (rate ratio 0.80 [95% CI: 0.63 to 1.03]; *P* = .084) with a similar result in a COVID-sensitivity analysis (rate ratio 0.67 (95% CI: 0.48–0.93); *P* = .016). Trends to reductions in cardiovascular and all-cause mortality for patients assigned to FDI were not significant.

Conversely, in the subset of patients (*n* = 269) with a TSAT ≥ 20% but ferritin < 100 µg/L, administration of FDI had only small effects on haemoglobin and MLwHF scores. This group of patients had a lower rate for the primary endpoint than those with a TSAT < 20%, with some evidence of benefit for those assigned to FDI in the first two years. However, a COVID-sensitivity analysis (*Table 6*) did not identify greater benefit and all-cause mortality tended to be higher for patients assigned to FDI for patients with a TSAT ≥ 20% but ferritin < 100 µg/L.

**Table 6:** Effects of ferric derisomaltose compared to usual care for patients classified by serum ferritin concentration and transferrin saturation

TSAT	≥20%		≥20%		<20%		<20%		<20%		*P* for interaction
Serum ferritin concentration	≤30 µg/L		>30 to <100 µg/L		≤30 µg/L		>30 to <100 µg/L		≥100 µg/L		
	Usual care	FDI	Usual care	FDI	Usual care	FDI	Usual care	FDI	Usual care	FDI	
	21	25	107	116	122	120	203	185	101*	108	
Haemoglobin (g/dL)											
Value at 4 months	12.8 (1.7)	13.0 (1.0)	12.4 (1.3)	12.7 (1.1)	12.4 (1.5)	13.5 (1.4)	12.1 (1.5)	12.7 (1.4)	12.0 (1.4)	12.3 (1.4)	
Difference in change from baseline	0.3 (−0.4 to 1.0)		0.3 (0.1 to 0.6)		1.1 (0.7 to 1.5)		0.6 (0.3 to 0.9)		0.5 (0.1 to 0.8)		.008
MLwHF score overall (*n*)	20	25	95	110	106	103	164	160	90	91	
Value at 4 months	41 (22)	30 (21)	34 (25)	34 (24)	39 (26)	38 (28)	42 (27)	38 (29)	42 (27)	39 (28)	
Difference at 4 months	− 11 (−24 to +2)		−1 (−7 to +6)		−2 (−9 to +6)		−4 (−10 to +2)		−3 (−11 to +5)		.79
MLwHF score physical	20	25									
Value at 4 months	19 (10)	15 (9)	18 (11)	18 (11)	20 (12)	18 (12)	21 (12)	19 (12)	21 (12)	19 (12)	
Difference at 4 months	−4 (−10 to +2)		−0 (−3 to +3)		−2 (−6 to +1)		−2 (−5 to +0)		−1 (−5 to +2)		.50
MLwHF score emotional											
Value at 4 months	11 (7)	6 (6)	7 (8)	7 (7)	8 (8)	10 (8)	10 (8)	8 (8)	9 (7)	9 (8)	
Difference at 4 months	−4 (−8 to −1)		−0 (−2 to +2)		+1 (−1 to +3)		−1 (−3 to +1)		−1 (−3 to +2)		.21
6-Minute walk test (*n*)	11	16	50	63	52	47	72	82	38	44	
Distance at 4 months (m)	349 (117)	301 (163)	319 (123)	296 (127)	282 (119)	280 (134)	270 (145)	271 (133)	240 (116)	280 (122)	
Difference usual care vs. FDI	−47 (−165 to +71)		−23 (−70 to +24)		−2 (−52 to +48)		+1 (−44 to +45)		+40 (−12 to +93)		.45
Primary endpoint											
Rate per 100 patient-years	8.7	10.8	22.9	19.4	19.9	13.1	30.5	28.9	39.6	26.9	
Rate ratio	1.64 (0.38 to 7.09)		0.92 (0.57 to 1.49)		0.65 (0.38 to 1.13)		0.95 (0.68 to 1.33)		0.67 (0.42 to 1.07)		.69
Absolute difference in rate	2.1 higher for FDI		3.5 lower for FDI		**6.8 lower for FDI**		1.6 lower for FDI		**12.7 lower for FDI**		
Primary endpoint (COVID sensitivity)											
Rate per 100 patient-years	7.0	6.2	21.4	20.1	18.8	11.6	34.1	29.9	44.1	24.3	
Rate ratio	Not calculated		0.97 (0.54 to 1.74)		0.62 (0.32 to 1.20)		0.89 (0.59 to 1.33)		0.53 (0.29 to 0.98)		.62
Absolute difference in rate	Not calculated		1.3 lower for FDI		**7.2 lower for FDI**		4.2 lower for FDI		**19.8 lower for FDI**		
CV mortality											
Percentage (%)	4.8	12.0	21.5	19.0	15.6	13.3	30.5	27.6	26.7	20.4	
Hazard ratio	Not calculated		0.94 (0.52 to 1.70)		0.88 (0.45 to 1.71)		0.88 (0.61 to 1.27)		0.72 (0.41 to 1.27)		.81
Absolute difference in percentages (%)	Not calculated		2.5 lower for FDI		2.3 lower for FDI		2.9 lower for FDI		**6.3 lower for FDI**		
CV mortality (COVID sensitivity)											
Percentage (%)	0.0	8.0	14.0	12.7	6.8	8.1	20.8	15.5	20.2	11.0	
Hazard ratio	Not calculated		0.98 (0.47 to 3.19)		1.23 (0.47 to 3.19)		0.68 (0.42 to 1.12)		0.55 (0.26 to 1.16)		.70
Absolute difference in percentages (%)	Not calculated		1.3 lower for FDI		1.3 higher for FDI		**5.3 lower for FDI**		**9.2 lower for FDI**		
All-cause mortality											
Percentage (%)	9.5	28.0	29.0	31.0	27.0	26.7	38.9	39.5	39.6	27.8	
Hazard ratio	Not calculated		1.16 (0.72 to 1.89)		1.04 (0.64 to 1.70)		0.98 (0.72 to 1.35)		0.67 (0.42 to 1.07)		.25
Absolute difference in percentages (%)	Not calculated		2.0 higher for FDI		0.3 lower for FDI		0.6 higher for FDI		**11.8 lower for FDI**		
All-cause mortality (COVID sensitivity)											
Percentage (%)	5.6	16.0	16.0	20.9	13.6	17.1	27.1	22.0	25.5	15.0	
Hazard ratio	Not calculated		1.43 (0.75 to 2.71)		1.27 (0.65 to 2.48)		0.75 (0.49 to 1.14)		0.58 (0.31 to 1.12)		.16
Absolute difference in percentages (%)	Not calculated		4.9 higher for FDI		3.5 higher for FDI		**5.1 lower for FDI**		**10.5 lower for FDI**		

Substantial differences in rates are highlighted in bold. MLwHF, Minnesota Living with Heart Failure. 101* - one patient with a serum ferritin ≥100 µg/L did not have a measurement of TSAT accounting for differences from Table 3.

### Analysis by ferritin and TSAT

Further analysis classifying patients by TSAT < 20% or ≥20% and each of the three ferritin categories found only a few patients with a TSAT ≥ 20% and a ferritin ≤ 30 µg/L (*n* = 46) who showed little increase in haemoglobin after administration of FDI and had low rates of clinical events with usual care that were not reduced by FDI (see Supplementary data online, *Table S1B*; *Table 6*). For patients with a TSAT < 20%, those with a serum ferritin ≤ 30 µg/L had a similar prevalence of anaemia compared to those with a higher ferritin, but a larger increase in haemoglobin after receiving FDI, achieving a higher haemoglobin by 4 months. For patients with a TSAT < 20%, the higher the ferritin, the greater the rate of events and, although there was no statistical interaction between TSAT/ferritin categories, the largest absolute differences were observed in those with a TSAT < 20% and a ferritin ≥ 100 µg/L (*Table 6*, *Figures 4* and *5*).

## Discussion

An intervention designed to fix a problem that does not exist is unlikely to succeed. Giving patients who are not iron deficient more iron may not be helpful and could be harmful.^17^ This analysis suggests that either anaemia severity or a TSAT < 20% might identify patients with heart failure who are most likely to benefit from i.v. iron; the relationship between serum ferritin and response to i.v. FDI was complex and appeared of little practical clinical value (*Structured Graphical Abstract*). Few patients fulfilled WHO criteria for iron deficiency; a serum ferritin < 15 µg/dL.

Patients with moderately severe anaemia had a poorer quality of life and prognosis than patients without anaemia and responded to administration of i.v. FDI with a substantial increase in haemoglobin and improvement in quality of life. Although, in relative terms, the effect of FDI on clinical event rates was similar regardless of anaemia severity, because patients with more severe anaemia had higher event rates, the absolute benefit was greatest for this group. Correction of anaemia might be an important mechanism by which i.v. iron delivers benefit, but the severity of anaemia might also serve as a marker of iron deficiency affecting many other metabolic processes. Trials of i.v. ferric carboxymaltose (FCM) suggest that haemoglobin does not predict a greater response to iron supplements,^18,19^ but this may reflect differences in the definition of anaemia used, the severity of congestion, which may affect plasma volume and therefore haemoglobin concentration, and the duration of follow-up.

A low serum ferritin and a low TSAT are both considered to be markers of iron deficiency. However, for patients with cardiovascular disease, there is a strong U-shaped relationship between TSAT and prognosis, with the nadir of risk lying between 30% and 39% but, in contrast, a lower serum ferritin is associated with a better prognosis.^11^ This apparent paradox requires explanation. In IRONMAN, a serum ferritin ≤ 30 µg/L was associated with features suggesting less severe heart failure and a better prognosis, including milder symptoms, lower NT-proBNP, and better renal function, therefore it is not surprising that, consistent with findings from previous observational studies of heart failure,^6,9^ ferritin was associated with a low rate for the primary endpoint whether or not patients were randomized to i.v. iron. Ferritin < 100 µg/L, unless associated with a low TSAT, was also a poor predictor of the response to i.v. iron by any of the criteria we used. Interestingly, 84% of patients with a ferritin ≤ 30 µg/L had a TSAT < 20%. These patients had a substantial increase in haemoglobin in response to i.v. iron and, despite a relatively good prognosis, appeared to benefit further from i.v. iron. Ferritin is a large molecule that can only escape from cells by exocytosis or cell death. Inflammation may increase the rate of exocytosis and macrophage cell death leading to increases in serum ferritin.^17^ Accordingly, a low serum ferritin may require both iron deficiency and good cell health, explaining why patients who have both a low ferritin and a low TSAT have a relatively good prognosis but still respond to iron. In contrast, patients with a higher serum ferritin but TSAT < 20% had a high rate of events in the control group but, in absolute terms, a substantial reduction in events after i.v. iron. In these cases, a normal serum ferritin may reflect cellular damage and death, which conceals iron deficiency in patients with more advanced disease.

The proportion of patients with anaemia increased progressively as TSAT declined and the increase in haemoglobin after administration of FDI was most striking when TSAT was ≤10% or <20% with serum ferritin ≤ 30 µg/L, which often led to correction of anaemia. Patients with a TSAT < 20% and serum ferritin > 30 µg/L were more likely to remain anaemic after administration of FDI, suggesting that factors other than iron deficiency, such as inflammation and erythropoietin deficiency or resistance, contributed to their anaemia.^6^ Despite the strong relationship between TSAT and the increase in haemoglobin with FDI, TSAT did not predict the effects of FDI on symptoms or walk distance. Patients with a TSAT < 20% had a higher rate of clinical events and tended to have greater relative reductions in event rates if randomized to FDI and, consequently, they had a greater absolute reduction in events, although tests for interaction were not statistically significant. In contrast, patients with a TSAT ≥ 20% (with serum ferritin < 100 µg/L) appeared to receive little benefit from i.v. FDI in terms of haemoglobin response, symptoms, walk distance, or reduction in clinical events.

It is not clear that measurement of serum ferritin is useful for identifying patients with heart failure who benefit from i.v. iron, and it may cause confusion and uncertainty in clinical practice. It is widely believed that when serum ferritin and TSAT are both low, this reflects absolute iron deficiency and that a normal or raised serum ferritin with a low TSAT reflects functional iron deficiency, meaning that iron is trapped in the liver and reticulo-endothelial system and is no longer available for metabolic requirements.^7,12^ However, it is difficult to prove that a patient has functional iron deficiency based on measurement of serum ferritin and TSAT alone. Measurement of hepcidin or soluble transferrin receptors might help identify functional iron deficiency, but theoretical constructs require direct proof that intra-cellular ferritin is increased. If the goal of treatment is to improve symptoms or prognosis for patients with heart failure, focussing on those with anaemia and/or TSAT < 20% might be a good strategy for clinical practice.

Recently, the HEART-FID trial found little evidence that i.v. FCM improved either hospitalization for heart failure or mortality.^20^ However, at baseline, 60% of patients had a TSAT of 20% or more and the mean haemoglobin was 12.6 g/dL, suggesting that many had neither iron deficiency nor anaemia. A meta-analysis of individual-patient data (IPD) from trials of FCM and aggregate data from IRONMAN did not provide conclusive evidence that i.v. iron substantially reduced the composite outcomes of cardiovascular hospitalizations or death (rate ratio 0.86 [95% CI 0.75 to 0.98]) or heart failure hospitalization or cardiovascular death (rate ratio 0.87 [95% CI 0.75 to 1.01]).^21^ However, the IPD meta-analysis found a strong interaction between TSAT and the reduction in these outcomes and in cardiovascular death. Benefits were substantial benefit when TSAT was <15% but there were trends to harm when TSAT was ≥24%. A reasonable interpretation of the totality of evidence is that many patients enrolled in randomized trials could not benefit from i.v. or oral iron supplements because they did not have iron deficiency but that patients who do have iron deficiency, as evidenced by a low TSAT, might benefit substantially.

Although highly correlated with TSAT, serum iron might be the better marker of prognosis for patients with cardiovascular disease.^1,9,22^ TSAT depends not only on serum concentrations of iron but also of transferrin, which is inversely related to serum ferritin.^5^ As serum ferritin rises, transferrin falls, leading to a higher TSAT for a given serum iron concentration. Conversely, when serum ferritin is low, transferrin will be higher, leading to lower TSAT for a given serum iron concentration. If serum iron is a better marker of iron deficiency than TSAT, this might explain the lack of a clear relationship between decrements in TSAT below 20% and the magnitude of response to FDI. Patients with a serum ferritin > 400 µg/L were excluded from IRONMAN. In retrospect, measurements of TSAT or serum iron may be better ways of excluding iron overload, again making measurement of serum ferritin clinically redundant. Other markers of iron deficiency might also be considered for the identification of patients with iron deficiency more likely to benefit from iron supplements, but they have not been studied in randomized trials.^6^

There are many limitations to this analysis. Pre-specified analyses investigating interactions between assigned treatment and markers of iron deficiency showed only trends rather than statistically significant differences, which was also true for most secondary outcomes. Most subgroups were small with insufficient power to confirm numerically striking differences. Ultimately, our observations and interpretation need to be tested in other datasets. The COVID pandemic prevented recruitment of the planned number of patients, few patients received i.v. FDI after the first lockdown in March 2020 and many patients in the control group received oral iron, all of which may have diminished the ability to discern any benefits conferred by i.v. iron.

Only 57% of patients had a 6-minute walk test recorded at 4 months for which there are various explanations. Patients were not required to be able and willing to do a walking test to be included in the trial. The protocol stated that the test was ‘not mandated but encouraged’. It was a secondary outcome. Staff may not have had the time or a suitable 30 m corridor free of obstructions to conduct the walking test. The COVID pandemic will have prevented some in-person visits. However, in the HEART-FID trial, despite including >3000 patients, there was little difference in 6-minute walk test distance between those who received placebo or FCM at 6 months.

Inclusion of patients in randomized trials required only a single measurement of haemoglobin and iron indices. However, all of these measures vary according to the severity of congestion, recent infection, inflammation, time of day, and from day to day.^23–25^ This might lead to incorrect classification of iron deficiency, further diluting the apparent benefits of i.v. iron. The diurnal variation observed in serum iron, with values peaking in the late morning, also applies to TSAT and possibly to ferritin.^23^ However, variations in blood markers during usual working hours (8 a.m. to 6 p.m.) are generally <10%; variations from 1 day to the next may be greater.^25^ Using two criteria to define the need for i.v. iron, for instance anaemia with a TSAT < 20%, might reduce misclassification.

In conclusion, based on the results of IRONMAN, there appears to be little value serum ferritin for selecting patients with heart failure and reduced ejection fraction likely to benefit from i.v. FDI. Patients with moderately severe anaemia, most of whom had a TSAT < 20%, were at higher risk of events and may have obtained more benefit from i.v. FDI in terms of symptoms and absolute reduction in clinical events than patients who were not anaemic. Patients with a TSAT < 20%, especially when serum ferritin was >100 µg/L, also appeared more likely to benefit in terms of absolute and relative reduction in clinical events. These results, if confirmed in other trials, could simplify guideline recommendations for the diagnosis of iron deficiency, thereby avoiding administration of i.v. iron to patients who have little to gain, targeting it at those most likely to benefit, while ensuring good use of health service resources and patients’ time and effort required to deliver therapy.

## Supplementary Material

ehae086_Supplementary_Data

## Data Availability

The IRONMAN investigators welcome proposals for data sharing after the publication of the primary study results and key secondary manuscripts (∼2 years after the publication of the primary results). Proposals will be considered by the IRONMAN Publications Committee. Approval will depend on the scientific value of the proposal, compatibility with the original patient consent, and data protection legislation. Preference will be given to proposals for access to aggregate data or analytic results. Applicants may be expected to meet the costs associated with the preparation of data or statistical analysis. Applications should be made to Paul Kalra (paulkalra@doctors.org.uk).

## Pre-registered Clinical Trial Number

IRONMAN is registered with ClinicalTrials.gov, NCT02642562.
