# Correction to: Intravenous iron for heart failure, iron deficiency definitions, and clinical response: the IRONMAN trial

**DOI:** 10.1093/eurheartj/ehae243

**Published:** 2024-08-20

This is a correction to: John G F Cleland, Philip A Kalra, Pierpaolo Pellicori, Fraser J Graham, Paul W X Foley, Iain B Squire, Peter J Cowburn, Alison Seed, Andrew L Clark, Ben Szwejkowski, Prithwish Banerjee, Justin Cooke, Mark Francis, Piers Clifford, Aaron Wong, Colin Petrie, John J V McMurray, Elizabeth A Thomson, Kirsty Wetherall, Michele Robertson, Ian Ford, Paul R Kalra, the IRONMAN Study Group, Intravenous iron for heart failure, iron deficiency definitions, and clinical response: the IRONMAN trial, *European Heart Journal*, Volume 45, Issue 16, 21 April 2024, Pages 1410–1426, https://doi.org/10.1093/eurheartj/ehae086.

The authors identified a problem with the software that generated the figures for the recurrent event rate analyses. The plots should represent Ghosh and Lin plots, which depict the expected number of events over time. The plots displayed in the original version of the paper submitted actually contain estimates of the probability over time that at least one event has been observed, a less interesting quantity. Accordingly, the Graphical Abstract and Figures 3, 4 and 5 have been updated. The results of the formal statistical analyses and the interpretation of the data are not affected by this error.

Additionally, the originally published version of the manuscript contained a number of errors in the labelling of tables. The following corrections have been made:

**Table 2**

In row 7, “Rate per patient-year” has been changed to “Rate per 100 patient-years.”

In rows 8-11, “Rate per 100 patient-years” has been changed to “Percentage (%),” and “Absolute Difference in Rate” has been changed to “Absolute Difference in Percentages (%).”

**Table 3**

In the column headers, “Control” has been changed to “Usual Care.”

In the right-hand column, the missing number “91” has been added.

In rows 7-11, the same corrections have been made as for Table 2.

The following note has been added to the table footnote: “One patient assigned to usual care did not have a measurement of TSAT but is included here.”

**Table 5**

In rows 7-11, the same corrections have been made as for Table 2.

**Table 6**

In the header in the right-hand column for Usual care, “102” has been changed to “101*”

The following footnote has been added: “101* - one patient with a serum ferritin ≥100 µg/L did not have a measurement of TSAT accounting for differences from Table 3.”

In rows 7-11, the same corrections have been made as for Table 2.

Various extraneous numbers of events, which were used to calculate rates, have also been removed.

