# Supplementary material for: Intravenous iron for heart failure, iron deficiency definitions, and clinical response: the IRONMAN trial
Source: Eur Heart J. 2024 Mar 6;45(16):1410–26. doi: 10.1093/eurheartj/ehae086 (PMC11032711; doi:10.1093/eurheartj/ehae086)
Supplement: ehae086_Supplementary_Data [file ehae086_supplementary_data.zip › IRONMAN Study Group Members 2023.docx]

IRONMAN STUDY GROUP

LIST OF INVESTIGATORS

Paul Kalra, Elena Cowan, Charlotte Turner, Rosalynn Austin, Queen Alexandra Hospital, Portsmouth, UK

Rebeca Lane, Paula Rogers, Royal Brompton and Harefield Hospital, London, UK

Paul Foley, Badri Chandrasekaran, Eva Fraile, Lynsey Kyeremeh, Great Western Hospital, Swindon, UK

Fozia Ahmed, Manchester Royal Infirmary, Manchester, UK

Mark Petrie, Lorraine McGregor, Joanna Osmanska, Fraser Graham, Glasgow Royal Infirmary, Glasgow, UK

Ninian Lang, Barbara Meyer, Faheem Ahmad, Joanna Osmanska, Queen Elizabeth University Hospital, Glasgow, UK

Iain Squire, Jude Fisher, Glenfield Hospital, Leicester, UK

Philip Kalra, Christina Summersgill, Katarzyna Adeniji, Rajkumar Chinnadurai, Salford Royal Hospital, Salford, UK

Andrew Ludman, Lisa Massimo, Clare Hardman, Daisy Sykes, Royal Devon and Exeter Hospital, Exeter, UK

Peter Cowburn, Sarah Frank, Simon Smith, University Hospital Southampton, Southampton, UK

Alan Japp, Mohamed Anwar, Beth Whittington, Royal Infirmary of Edinburgh, Edinburgh, UK

Alison Seed, Blackpool Victoria Hospital, Blackpool, UK

Robin Ray, Vennessa Sookhoo, Sinead Lyons, St. George’s Hospital, London, UK

Abdallah Al-Mohammad, Janet Middle, Kay Housley, Northern General Hospital, Sheffield, UK

Andrew Clark, Jeanne Bulemfu, Castle Hill Hospital, Hull, UK

Christopher Critoph, Royal Bournemouth Hospital, Bournemouth, UK

Victor Chong, Stephen Wood, University Hospital Crosshouse, Kilmarnock, UK

Benjamin Szwejkowski, Chim Lang, Jackie Duff, Susan MacDonald, Ninewells Hospital, Dundee, UK

Rebekah Schiff, Guy’s and St Thomas’ Hospital, London, UK

Patrick Donnelly, Ulster Hospital, Dundonald, UK

Thuraia Nageh, Swapna Kunhunny, Southend University Hospital, Southend, UK

Mark Petrie, Roy Gardner, Marion McAdam, Elizabeth McPherson, Golden Jubilee National Hospital, Clydebank, UK

Prithwish Banerjee, Eleanor Sear, Nigel Edwards, University Hospital Coventry, Coventry, UK

Jason Glover, Basingstoke and North Hampshire Hospital, Basingstoke, UK

Pierpaolo Pellicori, Clare Murphy, Royal Alexandra Hospital, Paisley, UK

Justin Cooke, Chesterfield Royal Hospital, Chesterfield, UK

Charles Spencer, New Cross Hospital, Wolverhampton, UK

Mark Francis, Victoria Hospital, Kirkcaldy, UK

Iain Matthews, Hayley McKie, Wansbeck General Hospital, UK

Andrew Marshall, Janet Large, Jenny Stratford, District General Hospital, Eastbourne, UK

Piers Clifford, Sara Tavares, Hammersmith Hospital, London, UK

Christopher Boos, Poole Hospital, Poole, UK

Philip Keeling, Debbie Hughes, Torbay Hospital, Torquay, UK

Aaron Wong, Deborah Jones, Alex James, Rhys Williams, Princess of Wales Hospital, Bridgend, UK

Stephen Leslie, Jim Finlayson, Raigmore Hospital, Inverness, UK

Piers Clifford, Wycombe Hospital, High Wycombe, UK

Andrew Hannah, Aberdeen Royal Infirmary, Aberdeen, UK

Philip Campbell, Royal Gwent Hospital, Newport, UK

John Walsh, Jane Quinn, Nottingham University Hospital, Nottingham, UK

Callum Chapman, West Middlesex University Hospital, Isleworth, UK

Susan Piper, Sheetal Patale, King’s College Hospital, London, UK

Preeti Gupta, Victor Sim, Lucy Knibbs, University Hospital Llandough, Penarth, UK

Kristopher Lyons, Antrim Area Hospital, Antrim, UK

Lana Dixon, Royal Victoria Hospital, Belfast, UK

Colin Petrie, University Hospital Monklands, Airdrie, UK

Yuk-ki Wong, St Richard’s Hospital, Chichester, UK

Catherine Labinjoh, Forth Valley Royal Hospital, Larbert, UK

Simon Duckett, Ian Massey, Royal Stoke University Hospital, Stoke-On-Trent, UK

Henry Savage, Sofia Matias, Jonaifah Ramirez, Basildon University Hospital, Basildon, UK

Charlotte Manisty, Ifza Hussain, St Bartholomew’s Hospital, London, UK

Rajiv Sankaranarayanan, Gershan Davis, Aintree University Hospital, Liverpool, UK

Samuel McClure, John Baxter, Sunderland Royal Hospital, Sunderland, UK

Eleanor Wicks, John Radcliffe Hospital, Oxford, UK

Jolanta Sobolewska, Royal Oldham Hospital, Oldham, UK

Jerry Murphy, Darlington Memorial Hospital, Darlington, UK

Ahmed Elzayat, Alastair Cooke, Doncaster Royal Infirmary, Doncaster, UK

Jay Wright, Liverpool Heart and Chest Hospital, Liverpool, UK

Simon Williams, Wythenshawe Hospital, Manchester, UK

Amal Muthumala, North Middlesex University Hospital, London, UK

Parminder Chaggar, Sue Webber, Royal Cornwall Hospital, Truro, UK

Gethin Ellis, Mandie Welch, Royal Glamorgan Hospital, Llantrisant, UK

Sudantha Bulugahapitiya, Bradford Royal Infirmary, Bradford, UK

Thomas Jackson, Salisbury District Hospital, Salisbury, UK

Tapesh Pakrashi, Kingston Hospital, Kingston Upon Thames, UK

Ameet Bakhai, Vinodh Krishnamurthy, Barnet Hospital, Barnet, UK

Reto Gamma, Broomfield Hospital, Chelmsford, UK

Susan Ellery, Royal Sussex County Hospital, Brighton, UK

Charlotte Manisty, University College London Hospital, London, UK

Geraint Jenkins, Gladdys Thomas, Morriston Hospital, Swansea, UK

Angus Nightingale, Bristol Royal Infirmary, Bristol, UK

CLINICAL TRIALS UNIT AND DATA AND STATISTICAL CENTRE

*Robertson Centre for Biostatistics, University of Glasgow*: Elizabeth Thomson (Project Management), Prof Ian Ford (Study Director), Michele Robertson, Nicola Greenlaw and Kirsty Wetherall (Biostatistics), Ross Clarke, Christopher Graham, Sharon Kean, Alan Stevenson and Robbie Wilson (eCRF), Sarah Boyle and John McHugh (Data Management), Lisa Hall and Joanne Woollard (Projects Administration), Claire Brunton, Dr Eleanor Dinnett and Amanda Reid (Endpoints Coordination)

NURSING AND ADMINISTRATIVE SUPPORT

Serena Howe, Portsmouth Hospitals University NHS Trust, Portsmouth, UK

Jill Nicholls, NHS Tayside, Dundee, UK

Anna Cunnington, Portsmouth Hospitals University NHS Trust, Portsmouth, UK

SPONSOR SUPPORT

From NHS Greater Glasgow and Clyde, Glasgow, UK: Dr Elizabeth Douglas, Dr Margaret Fegen, Dr Marc Jones, Dr Sheila McGowan, Dr Barbara Ross, Dr Pamela Sandu, Pamela Surtees

From University of Glasgow, Glasgow, UK: Dr Debra Stuart

STEERING COMMITTEE

Dr Nicholas Boon (Chair), Independent Member

Dr Shannon Amoils, British Heart Foundation, London, UK, non-voting

Dr Callum Chapman, Chelsea and Westminster Hospital NHS Foundation Trust, London, UK, Co-applicant

Prof John Cleland, University of Glasgow, Glasgow, UK, Co-applicant

Dr Thomas Goldin Diness, Pharmacosmos A/S, Holbaek, Denmark, non-voting

Prof Ian Ford, University of Glasgow, Glasgow, UK, Study Director

Prof Paul Kalra, Portsmouth Hospitals University NHS Trust, Portsmouth, UK, Chief Investigator

Prof Philip Kalra, Salford Royal Hospital, Northern Care Alliance NHS Foundation Trust, Salford, UK, Co-applicant

Prof Iain Macdougall, King’s College Hospital, London, UK, Co-applicant

Prof John McMurray, University of Glasgow, Glasgow, UK, Independent Member

Mr Richard Mindham, Independent Member, Patient representative

Prof Mark Petrie, University of Glasgow, Glasgow, UK, Co-applicant

Dr Pamela Sandu, NHS Greater Glasgow and Clyde, Glasgow, UK, Sponsor Representative, non-voting

Prof Iain Squire, University of Leicester, Leicester, UK, Co-applicant

Dr Claes Christian Strom, Pharmacosmos A/S, Holbaek, Denmark, non-voting (past member)

Ms Elizabeth Thomson, Project Manager, University of Glasgow, Glasgow, UK, non-voting

Dr Maureen Travers, NHS Greater Glasgow and Clyde, Glasgow, UK Sponsor Representative, non-voting (past member)

Prof Robert Wilcox, Independent Member

INDEPENDENT DATA MONITORING COMMITTEE

Prof Allan Struthers (Chair), University of Dundee, Dundee, UK

Prof Patrick Mark, University of Glasgow, Glasgow, UK

Prof Christopher Weir, University of Edinburgh, Edinburgh, UK

CLINICAL EVENT ADJUDICATION COMMITTEE

Prof John Cleland, University of Glasgow, Glasgow, UK

Dr Fraser Graham, University of Glasgow, Glasgow, UK

Dr Pierpaolo Pellicori, University of Glasgow, Glasgow, UK
