# Supplementary material for: Intravenous iron for heart failure, iron deficiency definitions, and clinical response: the IRONMAN trial
Source: Eur Heart J. 2024 Mar 6;45(16):1410–26. doi: 10.1093/eurheartj/ehae086 (PMC11032711; doi:10.1093/eurheartj/ehae086)
Supplement: ehae086_Supplementary_Data [file ehae086_supplementary_data.zip › IRONMAN TSAT Baseline Supp Table 1a_2023_11_01.docx]

**Baseline characteristics according to ferritin category**

| Ferritin | ≤30 µg/L | >30 to <100 µg/L | ≥100 µg/L | P = |
| --- | --- | --- | --- | --- |
| Total Number | 297 | 627 | 210 |  |
| Age | 73 [66, 79] | 74 [68, 80] | 73 [65, 78] | 0.13 |
| Women | 94 (32%) | 159 (25%) | 44 (21%) | 0.02 |
| Men | 203 (69%) | 468 (75%) | 166 (79%) |  |
| BMI kg/m^2^ | 28.7 [24.6, 32.7] | 27.9 [25.7, 32.0] | 29.1 [25.7, 33.1] | 0.02 |
| Medical history |  |  |  |  |
| Hypertension | 157 (53%) | 334 (53%) | 118 (56%) | 0.72 |
| Diabetes | 153 (52%) | 278 (44%) | 88 (42%) | 0.057 |
| Atrial Fibrillation | 126 (42%) | 305 (49%) | 102 (49%) | 0.18 |
| Aetiology |  |  |  |  |
| Ischaemic | 163 (55%) | 369 (59%) | 113 (54%) | 0.66 |
| Non-ischaemic | 101 (34%) | 196 (31%) | 75 (36%) |  |
| Unknown | 33 (11%) | 62 (10%) | 22 (11%) |  |
| Recruitment context |  |  |  |  |
| In-patient | 29 (10%) | 84 (13%) | 49 (23%) | 0.00039 |
| Discharged <6 months | 52 (18%) | 118 (19%) | 38 (18%) |  |
| Out-Patient | 216 (73%) | 425 (68%) | 123 (59%) |  |
| NYHA |  |  |  |  |
| II | 172 (58%) | 365 (58%) | 109 52%) | 0.008 |
| III/IV | 125 (42%) | 262 (42%) | 101 (48%) |  |
| Minnesota (n = ) | 293 | 614 | 204 |  |
| Overall | 44 [26, 62] | 42 [24, 61] | 45 [25, 67] | 0.33 |
| Physical | 24 [15, 31] | 23 [13, 31] | 24 [14, 32] | 0.87 |
| Emotional | 10 [3, 17] | 8 [2, 15] | 10 [4, 17] | 0.051 |
| 6-minute walk test (n = ) | 191 | 358 | 112 |  |
| Distance (m) | 275 [150, 355] | 270 [180, 355] | 269 [164, 342] | 0.70 |
| Physical Examination |  |  |  |  |
| Heart Rate beats/min | 68 (60, 78) | 69 (61, 79) | 71 (63, 80) | 0.09 |
| Systolic BP mmHg | 121 (109, 135) | 117 (105, 132) | 120 (107, 132) | 0.16 |
| Laboratory Tests |  |  |  |  |
| LVEF (%) | 33 (27, 37) | 34 (25, 38) | 33 (25, 38) | 0.77 |
| NT-proBNP (ng/L) | 1240 (639, 2483) | 1858 (991, 3833) | 2018 (117, 5246) | <0.0001 |
| eGFR (ml/min/1·73m^2^) | 56 (44, 79) | 51 (37, 66) | 46 (36, 64) | <0.0001 |
| Haemoglobin g/dL | 12.0 (11.1, 12.7) | 12.2 (11.3, 12.9) | 11.9 (10.8, 12.8) | 0.003 |
| No anaemia | 77 (26%) | 226 (36%) | 62 (30%) | 0.0032 |
| Mild anaemia | 108 (36%) | 185 (30%) | 55 (26%) |  |
| Moderate anaemia | 112 (38%) | 216 (34%) | 93 (44%) |  |
| TSAT (%) | 12 (8, 17) | 17 (12, 23) | 16 (12, 18) | <0.0001 |
| TSAT <20% | 242 (84%) | 387 (63%) | 210 (100%) | ** |
| On oral iron | 33 (11%) | 87 (14%) | 50 (24%) | 0.0002 |
| Heart failure medication |  |  |  |  |
| Loop diuretic | 225 (76%) | 518 (83%) | 180 (86%) | 0.009 |
| ACEi, ARB or ARNi | 263 (89%) | 545 (87%) | 173 (82%) | 0.12 |
| Beta-blocker | 273 (92%) | 552 (88%) | 181 (86%) | 0.097 |
| MRA | 176 (59%) | 341 (54%) | 114 (54%) | 0.34 |
| Digoxin | 31 (10%) | 79 (13%) | 24 (11%) | 0.34 |
| Any hypoglycaemic agent | 144 (49%) | 242 (39%) | 74 (35%) | 0.0037 |
| Insulin | 47 (16%) | 92 (15%) | 40 (19%) | 0.32 |
| SGLT2 inhibitor | 14 (5%) | 12 (2%) | 3 (1%) | 0.022 |
| Device therapy |  |  |  |  |
| ICD | 47 (16%) | 88 (14%) | 28 (13%) | 0.53 |
| PPM | 23 (8%) | 29 (5%) | 14 (7%) |  |
| CRT-P | 24 (8%) | 41 (7%) | 12 (6%) |  |
| CRT-D | 43 (15%) | 93 (15%) | 29 (14%) |  |

Data are number and percent or median with 1st and 3rd quartiles.

** analysis inappropriate since TSAT <20% used to classify patients.
