# Supplementary material for: Intravenous iron for heart failure, iron deficiency definitions, and clinical response: the IRONMAN trial
Source: Eur Heart J. 2024 Mar 6;45(16):1410–26. doi: 10.1093/eurheartj/ehae086 (PMC11032711; doi:10.1093/eurheartj/ehae086)
Supplement: ehae086_Supplementary_Data [file ehae086_supplementary_data.zip › IRONMAN TSAT Baseline Supp Table 1b_2023_11_01.docx]

**Baseline characteristics according to serum ferritin and TSAT categories**

| TSAT (%) | ≥20% | ≥20% | <20% | <20% | <20% | P = |
| --- | --- | --- | --- | --- | --- | --- |
| Ferritin ug/L | ≤30 ug/L | >30 to <100 | ≤30 ug/L | >30 to <100 | ≥100 ug/L |  |
| Number | 46 | 223 | 242 | 387 | 210 |  |
| Age (years) | 73 [68, 78] | 74 [68, 80] | 72 [66, 79] | 74 [67, 79] | 73 [65, 78] | 0.23 |
| Women | 10 (22%) | 51 (23%) | 80 (33%) | 106 (27%) | 43 (21%) | 0.023 |
| Men | 36 (78%) | 172 (77%) | 162 (67%) | 282 (73%) | 166 (79%) |  |
| BMI (kg/m^2^ ) | 27.4 [24.5, 30.9] | 27.5 [24.6, 31.6] | 28.7 [24.9, 33.0] | 27.8 [24.4, 32.3] | 29.1 [25.7, 33.1] | 0.0078 |
| Medical history — no. (%) |  |  |  |  |  |  |
| Hypertension | 23 (50%) | 107 (48%) | 129 (53%) | 216 (56%) | 118 (56%) | 0.35 |
| Diabetes | 27 (59%) | 86 (39%) | 122 (50%) | 182 (47%) | 88 (42%) | 0.025 |
| Atrial Fibrillation | 12 (26%) | 104 (47%) | 109 (45%) | 189 (49%) | 102 (49%) | 0.058 |
| Aetiology |  |  |  |  |  |  |
| Ischaemic | 27 (59%) | 136 (61%) | 133 (55%) | 224 (58%) | 113 (54%) | 0.80 |
| Non-ischaemic | 16 (35%) | 63 (28%) | 80 (33%) | 127 (33%) | 75 (36%) |  |
| Unknown | 3 (6%) | 24 (11%) | 29 (12%) | 37 (9%) | 22 (11%) |  |
| Recruitment context |  |  |  |  |  |  |
| In-patient | 0 | 14 (6%) | 24 (10%) | 60 (16%) | 49 (23%) | <0.0001 |
| Discharged <6 months | 8 (17%) | 42 (19%) | 42 (17%) | 77 (20%) | 37 (18%) |  |
| Out-Patient | 38 (83%) | 167 (75%) | 176 (73%) | 251 (65%) | 123 (59%) |  |
| NYHA |  |  |  |  |  |  |
| II | 33 (72%) | 150 (67%) | 137 (57%) | 208 (54%) | 109 52%) | 0.00034 |
| III | 13 (28%) | 73 (33%) | 105 (43%) | 180 (46%) | 101 (48%) |  |
| Minnesota (n = ) | 46 | 223 | 242 | 388 | 209 |  |
| Overall | 44 [32, 59] | 35 [17, 56] | 44 [26, 62] | 45 [26, 61] | 45 [25, 67] | 0.0051 |
| Physical | 24 [14, 29] | 19 [10, 29] | 23 [14, 31] | 25 [14, 31] | 24 [14, 32] | 0.029 |
| Emotional | 11 [4, 17] | 6 [2, 14] | 9 [3, 16] | 8 [3, 15] | 10 [4, 17] | 0.04 |
| 6-minute walk test (n =) | 35 | 144 | 154 | 207 | 112 |  |
| Distance (m) | 315 [203, 371] | 296 [206, 363] | 270 [143, 353] | 257 [172, 348] | 269 [164, 342] | 0.041 |
| Vital Signs |  |  |  |  |  |  |
| Heart Rate (beats/min) | 61 [57, 72] | 65 [60, 75] | 69 [60, 79] | 70 [61, 80] | 71 [63, 80] | 0.00013 |
| Systolic BP (mmHg) | 124 [110, 136] | 118 [105, 131] | 121 [108, 132] | 117 [105, 133] | 120 (107, 132) | 0.36 |
| Laboratory Tests |  |  |  |  |  |  |
| LVEF (%) | 33 [29, 36] | 35 [28, 39] | 34 [26, 37] | 31 [25, 37] | 33 (25, 38) | 0.024 |
| NT-proBNP (ng/L) | 874  [606, 1335] | 1555  [936, 2892] | 1350  [685, 2601] | 2549  [1030, 4495] | 2018  (117, 5246) | <0.0001 |
| eGFR (ml/min/1·73m^2^ ) | 56 [45, 72] | 52 [39, 69] | 56 [43, 80] | 50 [36, 64] | 46 [36, 64] | <0.0001 |
| Haemoglobin (g/dL) | 12.8 [12.0, 13.1] | 12.6 [11.7, 13.2] | 11.9 [11.1, 12.6] | 12.0 [11.1, 12.8] | 11.9 [10.8, 12.8] | <0.0001 |
| No anaemia | 19 (41%) | 104 (47%) | 55 (23%) | 121 (31%) | 61 (29%) | <0.0001 |
| Mild anaemia | 20 (44%) | 62 (28%) | 86 (36%) | 117 (30%) | 55 (26%) |  |
| Moderate anaemia | 7 (15%) | 57 (26%) | 101 (42%) | 150 (39%) | 93 (45%) |  |
| On oral iron | 9 (20% | 31 (14%) | 22 (9%) | 54 (14%) | 50 (24%) | 0.00031 |
| Heart Failure Medicines |  |  |  |  |  |  |
| Loop diuretic | 31 (67%) | 173 (78%) | 186 (77%) | 332 (86%) | 180 (86%) | 0.0011 |
| ACEi, ARB or ARNi | 44 (96%) | 207 (93%) | 213 (88%) | 327 (84%) | 172 (82%) | 0.0024 |
| Beta-blocker | 40 (87%) | 199 (89%) | 224 (93%) | 340 (88%) | 181 (86%) | 0.23 |
| MRA | 33 (72%) | 131 (59%) | 138 (57%) | 205 (53%) | 114 (54%) | 0.12 |
| Digoxin | 1 (2%) | 27 (12%) | 28 (12%) | 50 (13%) | 24 (12%) | 0.33 |
| Any hypoglycaemic agent | 24 (52% | 72 (32%) | 116 (48%) | 163 (42%) | 74 (35%) | 0.0019 |
| Insulin | 6 (13%) | 21 (9%) | 41 (17%) | 69 (18%) | 40 (19%) | 0.039 |
| SGLT2 inhibitor | 2 (4%) | 2 (1%) | 12 (5%) | 9 (2%) | 3 (1%) | 0.042 |
| Device therapy |  |  |  |  |  |  |
| ICD | 5 (11%) | 29 (13%) | 42 (17%) | 57 (15%) | 28 (13%) | 0.095 |
| PPCM | 6 (13%) | 4 (2%) | 15 (6%) | 24 (6%) | 14 (7%) |  |
| CRT-P | 1 (2%) | 17 (8%) | 23 (10%) | 23 (6%) | 12 (6%) |  |
| CRT-D | 11 (24% | 34 (15%) | 31 (13%) | 56 (14%) | 29 (14%) |  |

Data are number and percent or median with 1st and 3rd quartiles.
